# Physiological Responses and Bout Analysis in Elite Kickboxers During International K1 Competitions

**DOI:** 10.3389/fphys.2021.691028

**Published:** 2021-07-16

**Authors:** Łukasz Rydzik, Marcin Maciejczyk, Wojciech Czarny, Andzej Kędra, Tadeusz Ambroży

**Affiliations:** ^1^Institute of Sports Sciences, University of Physical Education, Kraków, Poland; ^2^Department of Physiology and Biochemistry, Faculty of Physical Education and Sport, University of Physical Education, Kraków, Poland; ^3^College of Medical Sciences, Institute of Physical Culture Studies, University of Rzeszow, Rzeszów, Poland; ^4^Department of Sports Kinanthropology, Faculty of Sports, University of Presov, Presov, Slovakia

**Keywords:** sports, kickboxing, fight analysis, heart rate, lactate

## Abstract

**Background:** Kickboxing is a combat sport with various forms of competition. Kickboxing according to the K1 rules is one of the most interesting and quickly developing forms of kickboxing. According to the K1 rules, it is possible to use a variety of techniques with great force. The aim of this study was to investigate the physiological responses during a real sports fight and to perform a technical and tactical analysis of the kickboxing bout according to the K1 rules.

**Methods:** This study was conducted during two cycles of the international kickboxing league according to the K1 rules in a group of 15 elite athletes. The indicators of technical and tactical training were evaluated in real sports bout. Blood lactate (LA) levels and heart rate (HR) were measured during and after the bout.

**Results:** The efficiency of the attack was on average 59.3 ± 2.7, its effectiveness was 50.3 ± 10.01, and its activeness was 112.3 ± 29. The peak LA concentration was 14.6 ± 1.9 mmol/L. LA concentration did not decrease to baseline after 20 min of recovery.

**Conclusion:** A kickboxing bout was found to induce strong physiological stress for the participants. Reported HR and LA concentration show that the intensity of the fight was close to maximal, and anaerobic metabolism played an important role during a fight.

## Introduction

Kickboxing is a striking combat sport that is very dynamic and characterized by high intensity (Di Marino, 2018). It requires a variety of complex skills and tactical excellence. Kickboxers are classified by gender, body mass, and age (Kordi et al., 2009). Kickboxing is characterized by continuous changes in movement structure under undetermined conditions, with variable work intensity and load duration (Krupalija et al., 2010). Most studies of kickboxers have been based on full-contact competitions, where, according to the rules, low kicks and knee strikes are not allowed. Previous studies have dealt with the physique of athletes, physiological responses, and time-motion and technical-tactical analyses in kickboxing athletes during simulated fights and training (Ouergui et al., 2013a, 2014b, 2016, 2019).

It is worth noticing that a kickboxing bout engages all muscle groups, requires coordination, and activates both aerobic and anaerobic metabolism (Senduran and Mutlu, 2017). A sports fight in K1 kickboxing is characterized by acyclic exercise and frequent changes of fighting conditions, which require good coordination and agility. This affects the athletes holistically and comprehensively engages the entire body by activating all muscle groups. With constant changes of a situation during a bout, the athletes need to show a keen level of proprioception, fast reaction time (both simple and complex), good hand-eye coordination (Akman et al., 2018), and spatial orientation and react immediately to the actions of his or her opponent (Slimani et al., 2017b). Preferred efforts both during the bout and training are based on submaximal and maximal loads (Rydzik and Ambrozy, 2021). However, there are no data concerning physiological responses to an effort during a real sports fight.

The efficiency of kickboxing techniques is based on a keen sense of the possibility of striking once or more than once. The strike requires high anaerobic power, while exercise during all intervals between strikes is based on aerobic metabolism (Ambrozy et al., 2020). Deciding moments of a fight that precede its settling (i.e., supramaximal intensity efforts requiring high power, speed, or strength from the athlete) are based on the alactic anaerobic energy pathway, but a high level of aerobic capacity is required for quick recovery between dynamic anaerobic efforts. This was confirmed by Zabukovec and Tiidus, who showed that elite kickboxers demonstrate a high level of aerobic and anaerobic fitness along with the ability to produce high muscle power (Zabukovec and Tiidus, 1995). Also, Ouergui et al. claim that kickboxing training should be aimed at improving the anaerobic capacity of athletes (Ouergui et al., 2014a). Although some studies on kickboxing have addressed these problems (Slimani et al., 2017b), few studies have explored physiological characteristics and performance aspects in kickboxers concerning weight categories in different kickboxing styles. However, each striking combat sport has its own rules, weight divisions, competitive levels, specific techniques, and consequently, different variables contributing to the success (Slimani et al., 2017c). The K1 formula is an interesting and dynamically developing kickboxing style. In the K1 rules, it is allowed to use many more techniques and strikes with a great force (Rydzik et al., 2020). All punches and kicks are allowed without restrictions on a striking force. During a fight, competitors can use all of the following techniques: roundhouse kick, front kick, side kick, axe kick, knee kick, back fist, and all boxing techniques. Competitors in the amateur type of K1 are characterized by a high sports skill level. Most of the skills defined as fighting techniques are determined by the optimal level of general and special physical fitness. For example, hand strikes, which are the basic technical elements in K1, require a high level of speed, dynamic strength of the upper limbs, and coordination of the whole body. Kicks primarily require good joint mobility (flexibility) and dynamic strength of the lower limbs (Rydzik and Ambrozy, 2021). Learning technical skills is the basis for tactical actions during the fight. Tactical actions are skills that athletes acquire through training and competitive experience. Tactics refers to offensive actions (i.e., combinations, agility, and timing) and defensive actions (i.e., dodges, blocks, and counterattacks). Tactical actions cannot be performed without proper technique and constitute a specific type of skill that guarantees success during the competition (Poteryakhin et al., 2021). According to the World Association of Kickboxing Organizations (WAKO), a kickboxing bout should consist of three 2-min rounds with 1-min breaks.

A detailed analysis of the physiological responses of the fighter during a fight is a key training parameter (Ouergui et al., 2013b). Studies concerning physiological responses during a real sports fight are especially valuable because they allow for the assessment of the workload during a fight and making adjustments in training to better fit the requirements of the fight (Slimani et al., 2017b). In addition to studying the physiological responses during kickboxing bouts, the hormonal responses of the body have also been examined (Moreira et al., 2010). Slimani et al. (2017a) studied kickboxers during a bout by dividing the time structure into three phases (i.e., preparatory activity time, fighting time, and break time). Based on the determination of the effectiveness of the fight and the combination of techniques used in individual phases, it was proposed to adapt the training programs to the specific requirements of weight categories and gender of kickboxers in order to develop technical–tactical skills that increase the chances of victory (Slimani et al., 2017a).

Additionally, a detailed technical analysis of a sports fight provides important information concerning further training. Computing the indicators of technical and tactical preparation is the best method of the assessment of the level of the training in this aspect (Adam and Sterkowicz-Przybycień, 2018). It allows for training optimization and implementation of specific training methods and techniques aimed at maximizing the exercise capacity of athletes (Moreira et al., 2010). Studies that assessed the physiological responses during a real sports fight are rare but they provide much valuable information. To our knowledge, this is the first study to analyze the fight in the aspect of physiological stress during a real rather than simulated sports fight during a competition. The main aim of this study was to evaluate physiological responses to a real sports fight according to the K1 rules. An additional aim was to make a technical and tactical analysis of the bout according to the K1 rules, which will allow for the evaluation of the ability of athletes to succeed in a real fight.

## Materials and Methods

### Study Design

**Figure 1:**
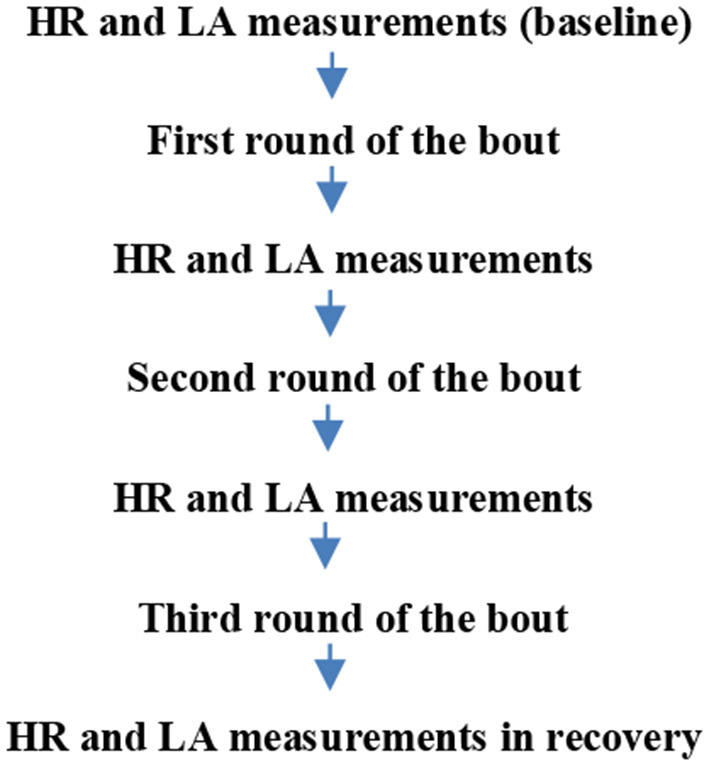
Study design.

This study was conducted during two cycles of the international kickboxing league according to the K1 rules in a group of 15 elite kickboxers. The inclusion criterion was the sports skill level determined based on the balance of fights won by the athlete. The measurements were done during a fight (consisting of three 2-min rounds) and following the fight. This study covered the technical analysis of the bout and measurements of lactate (LA) concentration and heart rate (HR). The following physiological measurements were taken: after the warm-up, when the athlete was ready to fight, directly after each round, and during recovery after the bout (Figure 1).

The fights were video recorded, and each recording was analyzed by an experienced coach for studied indicators (i.e., activeness, effectiveness, and efficiency of the attack). All participants provided their written consent to participate in the project. This study was approved by the Bioethical Committee at the Regional Medical Chamber in Kraków (No. 287/KBL/OIL/2020).

### Characteristics of the Participants

The mean age of the participants was 23.9 ± 4.6 years. The participants had practiced kickboxing for 9.9 ± 5.3 years on average (Table 1). Kickboxers were trained 5–6 times a week and were elite athletes who fought on average 20 kickboxing bouts a year. The somatic characteristics of the participants are presented in Table 1.

**Table 1:** Characteristics of the study group.

**Characteristics**	**$\bar{x}$**	**−95%Cl**	**+95%Cl**	**SD**	**Min**	**Max**	**Q1**	**Q3**
Age [ys]	23.9	21.4	26.5	4.6	19.0	34.0	20.0	26.0
Height[cm]	177.1	174.4	179.7	4.8	168.0	185.0	174.0	181.0
Body mass [kg]	79.1	75.9	82.2	5.6	67.0	86.0	75.0	84.0
BMI [kg/m^2^]	25.2	24.5	25.9	1.3	21.4	27.1	24.8	25.7
Training experience [ys]	9.9	6.9	12.8	5.3	4.0	25.0	6.0	11.0

### Analysis of the Fight

The analysis of the sports fight was performed by two champion-level kickboxing coaches and one referee based on digital video recordings of the examined athlete. The recording was made with three cameras. Movavi Video Editor 14 software (Movavi, Wildwood, MO, USA) was used to merge the images. The setting of cameras allowed continuous observation of the athletes, referees, and the scoreboard. A single sheet was developed as the essential research tool. Each expert had a separate measurement sheet on which they recorded the techniques used and those completed successfully. The data from all the measurement sheets were entered in an Excel sheet, where the means of the ratings of the three experts were computed for each athlete. Then the values of technical and tactical preparation indices were calculated. In the evaluation of the efficiency of the attack during K1 competitions, each fair hit gets 1 point. In the effectiveness of the attack, the effective attack is a technical action awarded a point, and an attack is defined as an offensive technical action. The indicators of technical and tactical training were computed using the following formulae (Rydzik et al., 2020; Rydzik and Ambrozy, 2021).

Efficiency of the attack (*S*_a_)

$$\begin{array}{l} S_{\text{a}}=\;\frac{n}{N} \end{array}$$

where *n* is the number of attacks awarded 1 pt.^*^ and *N* is the number of fights observed (it is 1 in this study).

Effectiveness of the attack (*E*_a_)

$$\begin{array}{l} E_{a}=\frac{number\;of\;efective\;attacks}{number\;of\;all\;attacks}\times \;100 \end{array}$$

Activeness of the attack (*A*_a_)

$$\begin{array}{l} A_{a}=\frac{number\;of\;all\;registered\;offensive\;actions\;of\;a\;kickboxer}{number\;of\;fights\;fought\;by\;a\;kickboxer\;\left(1\;in\;this\;study\right)} \end{array}$$

### Physiological Measurements

Heart rate measurement was performed using an HR monitor by Garmin Fenix 6 (Olathe, USA) with a chest strap. The first measurement was taken after a warm-up when a competitor was ready to fight. Subsequent measurements were taken directly after the first, second, and third rounds, and the final measurement was taken after the fight until the rest HR was equal to that when the athlete was ready to fight. The athletes were wearing the strap during 1-min breaks between rounds, and then the HR value was recorded. The measurements were taken during 1-min breaks between the rounds. Based on the received values of HR, a recovery index (RI) was calculated (Zatoń and Jastrzebska, 2010).

$$\begin{array}{l} RI=\frac{HR2-HR3}{HR2-HR1}\times \;100\% \end{array}$$

where HR1 is the baseline heart rate, HR2 is the peak heart rate noted immediately after the fight, and HR3 is the HR after 5 min of recovery.

Recovery index allows for the assessment of the efficiency of recovery. In the interpretation of RI, the following ranges were defined: <50%: poor recovery, 50–60%: average recovery, 60–80%: good recovery, and >80%: very good recovery.

The measurement of blood LA levels was performed with the use of Lactate Scout (Sweden) by taking a blood sample by finger stick. The first sample was obtained after the warm-up, and the subsequent samples were taken after each round of the fight during 1-min breaks between the rounds and after the fight in the 3rd and 20th minutes of recovery.

### Statistical Methods

The statistical analysis was conducted using Statistica 13.1 by StatSoft (Krakow, Poland). The data were tested for normal distribution using the Shapiro–Wilk test. ANOVA with repeated measures was used to compare the mean value of a variable in one measurement with the previous measurement. The homogeneity of variance within the groups was tested with the Levene's test. The Tukey's *post-hoc* test was also used, and the effect size was calculated. In basic descriptive statistics, arithmetic mean, standard deviation, minimal and maximal values, 95% CI, and first and third quartiles were used. The level of statistical significance was set at *p* < 0.05.

## Results

**Figure 2:**
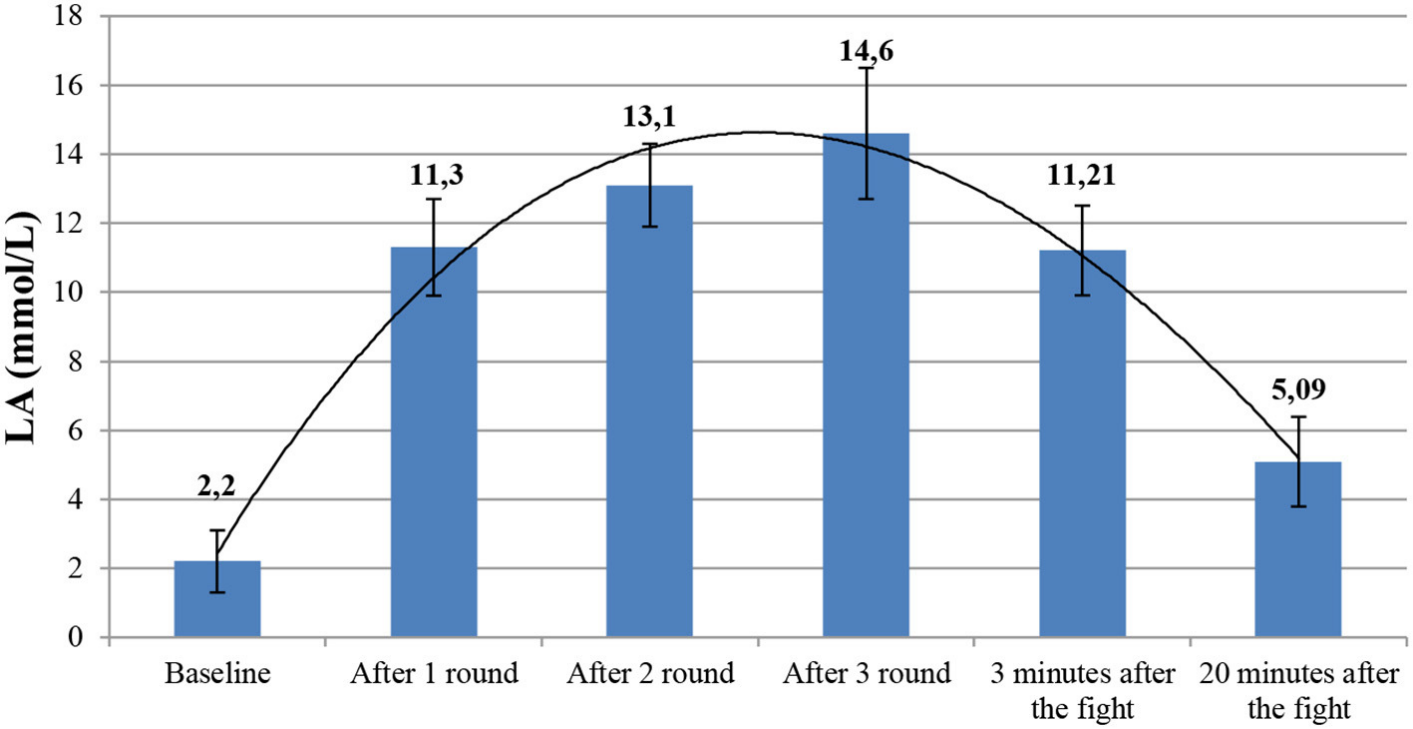
Lactate concentration in subsequent measurements.

**Figure 3:**
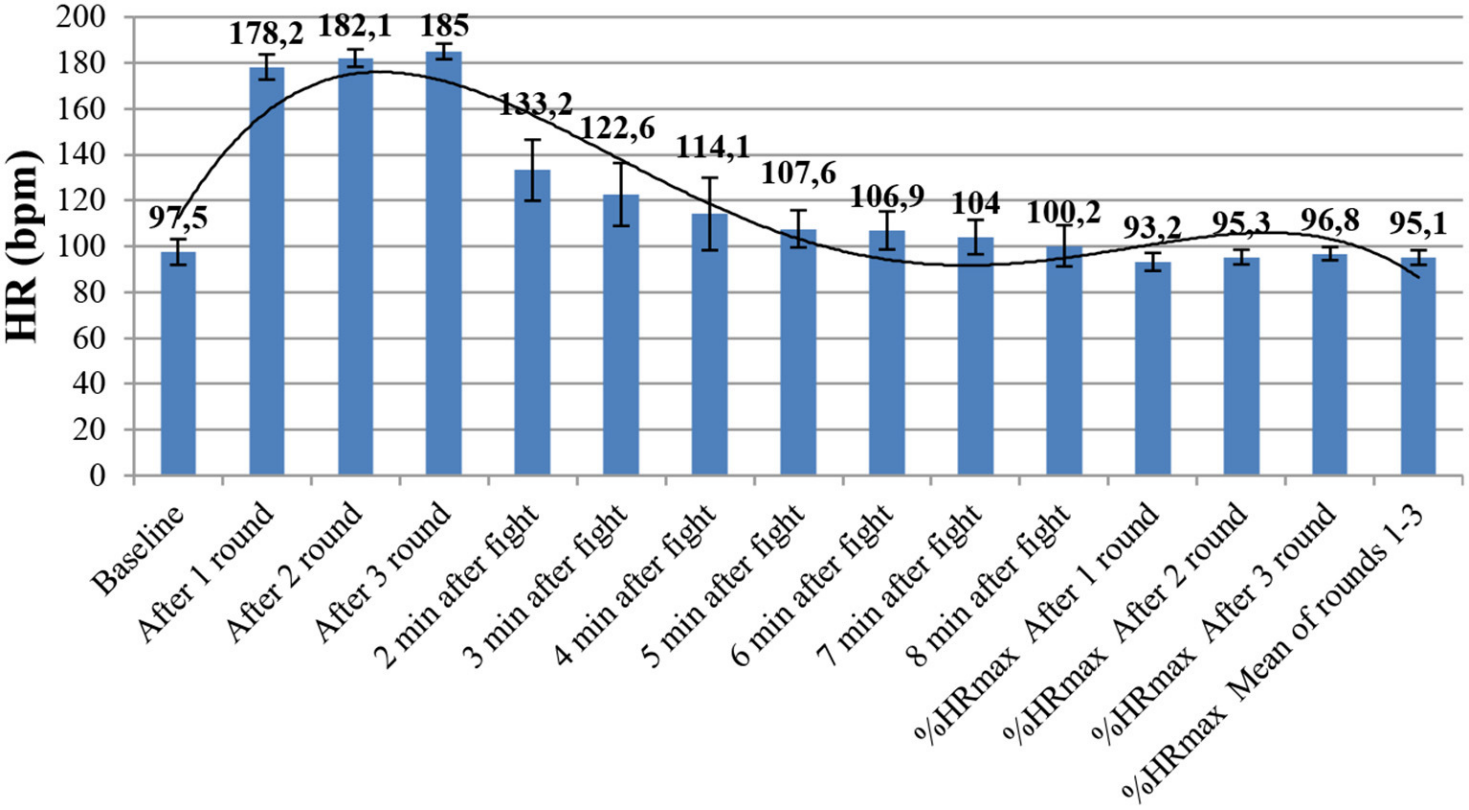
Heart rate in subsequent measurements.

The bouts induced strong physiological stress in the participants. A significant increase in the HR (*f* = 4,502.30 and *p* < 0.001) and blood LA (*f* = 26,425.45 and *p* < 0.001) were found during the fight. The peak LA concentration was noted in the third round (14.6 ± 1.9 mmol/L), and 20 min after the fight, it remained significantly higher compared with baseline (Table 2 and Figure 2). During the fight, the HRs were constantly increasing round after round and reached peak levels in the last round (185 ± 4.4 bpm). After the bout, the HR decreased to baseline after 5 min of recovery (Table 3 and Figure 3). The average RI in the fifth minute after the bout was 89.8% ± 10.4%. The average efficiency of the attack was 59.3 ± 2.7, its effectiveness was 50.3 ± 10.01, and activeness was 112.3 ± 29 (Table 4).

**Table 2:** Lactate concentration in subsequent measurements.

**La [mmol/L]**	**$\bar{x}$**	**−95%Cl**	**+95%Cl**	**SD**	**Min**	**Max**	**Q1**	**Q3**	**p_1_**	**p_2_**	**ES1**	**ES2**
Baseline	2.2	1.7	2.7	0.9	1.5	5.1	1.6	2.6	–	–	–	–
After 1 round	11.3	10.5	12.1	1.4	8.5	13.9	10.2	12.2	**<0.001**	**<0.001**	6.50	10.11
After 2 round	13.1	12.4	13.7	1.2	11.3	14.8	12.0	14.1	**<0.001**	**0.007**	9.08	1.29
After 3 round	14.6	13.6	15.7	1.9	10.0	16.9	13.4	16.2	**<0.001**	**0.024**	6.53	1.25
3 mins after fight	11.2	0.9	2.0	1.3	8.6	13.2	10.1	12.3	**<0.001**	**<0.001**	6.92	1.79
20 mins after fight	5.1	0.9	2.0	1.3	3.2	7.6	3.6	6.0	**<0.001**	**<0.001**	2.23	4.69
*p* (ANOVA)	<0.001											

*Statistically significant values are in bold*.

**Table 3:** Heart rate in subsequent measurements.

**HR**	**$\bar{x}$**	**−95%Cl**	**+95%Cl**	**SD**	**Min**	**Max**	**Q1**	**Q3**	**p_1_**	**p_2_**	**ES1**	**ES2**
Baseline	97.5	94.3	100.6	5.6	89.0	107.0	93.0	102.0	–	–	−	−
After 1 round	178.2	175.1	181.3	5.5	172.0	190.0	174.0	183.0	**<0.001**	**<0.001**	14.67	14.41
After 2 round	182.1	180.0	184.3	3.8	176.0	191.0	180.0	184.0	**<0.001**	0.989	22.26	0.71
After 3 round	185.0	183.1	186.9	3.4	180.0	192.0	183.0	188.0	**<0.001**	0.999	25.74	0.76
2 min after fight	133.2	125.8	140.6	13.3	117.0	162.0	120.0	138.0	**<0.001**	**<0.001**	2.68	15.24
3 min after fight	122.6	115.0	130.2	13.7	105.0	163.0	116.0	128.0	**<0.001**	0.084	1.83	0.80
4 min after fight	114.1	105.4	122.9	15.8	93.0	165.0	107.0	117.0	**<0.001**	0.348	1.05	0.62
5 min after fight	107.6	102.9	112.3	8.1	96.0	120.0	102.0	115.0	0.140	0.750	1.25	0.41
6 min after fight	106.9	101.3	112.5	8.3	96.0	122.0	99.0	114.0	0.309	1.000	1.13	0.09
7 min after fight	104.0	98.2	109.8	7.5	90.0	112.0	102.0	109.0	0.873	1.000	0.87	0.35
8 min after fight	100.2	93.3	107.1	9.0	88.0	111.0	90.0	106.0	1.000	0.999	0.30	0.51

*$\bar{x}$, mean; Min, minimum; Max, maximum; Q1, 1st quartile; Q3, 3rd quartile; SD, standard deviation*.

*p_1_–post-hoc: difference to baseline Tukey's test*.

*p_2_–post-hoc: difference between two consecutive measurements, Tukey's test*.

*ES1—effect size to baseline*.

*ES2—effect size between two consecutive measurements*.

*Statistically significant values are in bold*.

**Table 4:** Activeness, effectiveness, and efficiency of the attack.

	***n***	**$\bar{x}$**	**−95%Cl**	**+95%Cl**	**Min**	**Max**	**Q1**	**Q3**	**SD**
**Descriptive statistics**									
Efficiency	15	59.3	57.8	60.7	55.2	65.2	57.1	61.2	2.7
Effectiveness	15	50.3	44.7	56.0	31.0	65.0	41.0	61.0	10.1
Activeness	15	112.3	96.2	128.4	53.0	160.0	101.0	129.0	29.0

## Discussion

The objective of this study was to analyze the physiological responses during a real sports fight and to perform a technical and tactical analysis of the bout conducted according to the K1 rules. This study showed that a fight according to the K1 rules represented strong physiological stress for competitors. Blood LA levels after the bout exceeded 14 mmol/L. In this study, we used RI for the first time to analyze the physiological response. The recovery time, time, which should be as short as possible, can be an indirect indicator of aerobic capacity. A RI of ca. 90% indicates the high effectiveness of training used to develop aerobic capacity in kickboxers. Recovery time depends mostly on aerobic fitness and the ability to quickly recover, especially between the rounds. It is also essential for restoring the phosphagen level. Phosphagen, which is also restored through aerobic metabolism, is crucial for high-force kicks and punches. The data obtained in this study show that in order to meet the physiological demands in K1 formula, the competitors need comprehensive fitness training. To our knowledge, this study is the first to assess the physiological responses in this kickboxing style and can be compared only with different kickboxing formulae or different combat sports. A previous study (Ambrozy et al., 2020) showed that the performance of male kickboxers primarily depended on the alactic anaerobic and aerobic power, whereas our data also show that it is necessary for athletes to improve glycolytic anaerobic capacity, which was confirmed by a high LA concentration measured in the subsequent rounds of the bout. K1 formula is the most contact type of kickboxing and that can be a reason why the HR in this type of competition is usually higher than that in other kickboxing styles. Furthermore, the athletes often want to terminate the fight quickly in the first round and therefore they engage in the fight in the first round to a maximal level. In the K1 formula, athletes also perform the techniques at maximal intensity in order to terminate the fight quickly. Every technique performed by a kickboxer at maximal intensity causes an increased physiological response. In the study by Ghosh (2010), he presented the mean HR of boxers during a three-round bout (178 bpm). In contrast, during a 2-min contact karate bout, the HR was 160 bpm (Tabben et al., 2013). In this study, HR after 2 min of the fight was 18 bpm higher, which is likely to have resulted from a different character of the fight or a possibility of punching the head in K1 kickboxing, which is banned in kumite. A study of Turkish kickboxers showed the mean HR of 127 bpm, which is significantly lower than in fighters competing in K1 formula (Ambrozy et al., 2020). Both HR and exercise intensity were constantly rising in the subsequent rounds. It was observed that kickboxers experienced higher physiological stress and lower work outputs during consecutive rounds (Salci, 2015). Ouergui et al. (2019) observed a similar effect in their study. They recorded the highest increase in HR in the first round of the fight and the same increase in the third round. A similar round-to-round increase was observed in blood LA, which showed a gradual increase of glycolytic anaerobic potential during the bout. The mean LA concentration during the fight was close to that observed in Japanese semiprofessional K1 kickboxers (Ghosh, 2010) and higher than that observed in karate, taekwondo, and boxing (Ghosh, 2010; Tabben et al., 2013; Kim et al., 2014).

The achievement of the second aim of this study, which was to perform a technical and tactical analysis of the kickboxing bouts, is undoubtedly a novelty. Three parameters (i.e., the efficiency of the attack, the effectiveness of the attack, and the activeness of the attack) were used to assess the bouts. The level of activeness is measured by the number of techniques used (Ambrozy et al., 2021).

According to Laskowski et al. (2006), the far-reaching individualization of teaching technical and tactical skills should be an important element of the training process at the elite level. However, it seems appropriate to look for directions of changes within the sports fight depending on its physiological profile, which was the focus of this study.

Determination of the indicators of technical and tactical skills represents basic verification of the progress of athletes and helps compare him or her with other athletes. The indicators also allow for the identification of certain gaps in training. In this study, the level of the indicators of technical and tactical skills was at a high level compared with recent studies conducted by Ambrozy et al. (2021). All indicators were well above the average compared with the highest competitive disposition presented by the authors. This demonstrates that this study examined elite athletes, who at the time of measurement were characterized by an excellent competitive disposition. Previous study (Rydzik and Ambrozy, 2021) showed that elite kickboxers should be characterized by an ability to perform a wide range of tactical actions and optimally developed physical fitness. This level of preparation allows the athlete to quickly gain a point advantage, which may increase the likelihood of success (Boguszewski, 2014). The data show that in this kickboxing style (K1), the athletes require complex physical training to improve both aerobic and anaerobic capacities (Boguszewski, 2014).

### Limitation of This Study

The analysis of physiological responses during a sports fight is difficult and limits the study methodology and tools that can be used in a study. In this study, the measurements were limited to HR and blood LA, which are the only measurements possible to conduct during competition. As the measurements were taken during the real bout, the referees did not agree to wear the chest strap during the bout for safety reasons of athletes. Therefore, HR was measured by means of a chest strap that was put on the body of athletes immediately after each round of the fight. Unfortunately, we could not measure HRmax accurately, e.g., during a graded exercise test due to taking measurements during the real fight and express fight intensity as %HRmax. We used the highest recorded HR immediately after the bout to calculate the RI of HR. During the bout, HR increased steadily in subsequent rounds, but it is possible that during the third round, the HR was higher than immediately after the bout. However, for procedural reasons, it is impossible to measure HR during the fight.

In contrast, these are basic measurements that can be used to monitor training progress. Sports skill level or the fighting style of opponents can also influence the technical and tactical indicators or the physiological responses. Therefore, further research is needed to explore, e.g., physiological responses during a simulated fight, where more variables can be controlled.

## Conclusions

A fight was found to cause strong physiological stress for the fighters. Reported HR and LA concentration show that the intensity of work was close to maximal, and the anaerobic energy pathway was an important energy system contribution during a K1 fight. The data show that in this kickboxing style, athletes require comprehensive strength and conditioning programs to improve both aerobic and anaerobic capacities.

### Application

Efforts of the intensity close to maximal or supramaximal (i.e., alactic and lactic) should dominate in the training that prepares the kickboxers to competition according to the K1 rules. Using this type of effort in the training corresponds to the physiological requirements of the actual fight and should lead to the optimal fitness of a kickboxer.

## Data Availability Statement

The data presented in this study are available on request from the corresponding author.

## Ethics Statement

The studies involving human participants were reviewed and approved by The study was approved by the Bioethical Committee at the Regional Medical Chamber in Kraków (No. 287/KBL/OIL/2020). The patients/participants provided their written informed consent to participate in this study.

## Author Contributions

Conceptualization, Formal analysis, Investigation, Data curation, Writing—original draft preparation, Visualization, and Project administration: ŁR. Methodology: ŁR and MM. Software: AK and ŁR. Validation: ŁR and WC. Resources and Writing—review and editing: ŁR, MM, and TA. Supervision: TA and WC. Funding acquisition: MM. All authors contributed to the article and approved the submitted version.

## Conflict of Interest

The authors declare that the research was conducted in the absence of any commercial or financial relationships that could be construed as a potential conflict of interest.
